# Innovative composite tool use by Goffin’s cockatoos (*Cacatua goffiniana*)

**DOI:** 10.1038/s41598-022-05529-9

**Published:** 2022-01-27

**Authors:** Antonio J. Osuna-Mascaró, Roger Mundry, Sabine Tebbich, Sarah R. Beck, Alice M. I. Auersperg

**Affiliations:** 1 Messerli Research Institute, University of Veterinary Medicine, Medical University of Vienna and University of Vienna, Veterinärplatz 1, 1210 Vienna, Austria; 2 Department of Behavioural Biology, University of Vienna, Althanstraße 14, 1090 Vienna, Austria; 3 School of Psychology, University of Birmingham, Edgbaston, Birmingham, B15 2TT UK; 4 Present Address: German Primate Center, Leibniz Institute for Primate Research, Kellnerweg 4, 37077 Göttingen, Germany

**Keywords:** Animal behaviour, Zoology

## Abstract

Composite tool use (using more than one tool simultaneously to achieve an end) has played a significant role in the development of human technology. Typically, it depends on a number of specific and often complex spatial relations and there are thus very few reported cases in non-human animals (e.g., specific nut-cracking techniques in chimpanzees and capuchin monkeys). The innovative strategies underlying the innovation and spread of tool manufacture and associative tool use (using > 1 tools) across tool using animals is an important milestone towards a better understanding of the evolution of human technology. We tested Goffin’s cockatoos on a composite tool problem, the ‘Golf Club Task’, that requires the use of two objects in combination (one used to control the free movement of a second) to get a reward. We demonstrate that these parrots can innovate composite tool use by actively controlling the position of the end effector and movement of both objects involved in a goal directed manner. The consistent use of different techniques by different subjects highlights the innovative nature of the individual solutions. To test whether the solution could be socially transmitted, we conducted a second study, which provided only tentative evidence for emulative learning. To our knowledge, this indicates that the cognitive preconditions for composite tool use have also evolved outside the primate lineage.

## Introduction

Associative tool use (i.e., using more than one tool to achieve the same goal^1^), has played a significant role in human technical evolution. It includes, among other variants, compound and composite tool use. Both of the latter two types of associative tool use require an agent to use two or more tools at the same time, independent of one being attached to another. Prior to the innovation of compound tools for which the respective objects are combined (for example stuck or bound together) to achieve a single tool (e.g.^2,3^), some of the most successful tools of all time, were the result of combining the complementary function of two non-assembled objects. Such multiple tool constructs are referred to as ‘composite tools’^1,4^. For example, the sling and atlatl that humans have been using for tens of thousands of years^5,6^ are characterized by combining the complementary but distinct functions of two objects to achieve a single goal (in the case of the sling and the atlatl, to hit or stab a distant object/prey). Importantly, these actions are performed with two freely moving objects which at some stage form a stable structure. Humans also use tool composites in which the interaction between two or more objects is maintained in a constantly dynamic state. In modern times, the use of these tools has proliferated as recreational activities. A reason for this could be the level of precision required to succeed (from the traditional stickball of multiple ancestral cultures, to relatively modern sports such as tennis, baseball, polo or golf^7^).

In animals, however, composite tool use seems to be one of the least common types of tool use recorded so far^8^. The perhaps most studied case of composite tool use in nonhuman primates is nut cracking in chimpanzees of Bossou, Guinea^9,10^. An anvil and a hammer-stone, are used to crack nuts to gain access to the kernel^9–11^. Normally this is done by carrying a hammer stone to the place where a heavy anvil awaits, but at Bossou, chimpanzees often search for and place the anvil stone themselves^4^. Thus, anvil and hammer, following the most widespread terminology^1^, can be described as two tools whose functions are combined in a composite tool use. However according to the recent tooling framework proposed by Fragaszy and Mangalam^12^, the ungripped anvil would not be a tool per se (requires dynamic handling not a static placement).

Goffin's cockatoos are ideal non-primate models to study the origins of complex tool innovations. Despite not being dependent on tool obtained resources in the wild^13–15^, in captivity they have proven to be highly capable of innovating solutions to physical problems using tools^16–21^, and, as a recent discovery has shown, some wild individuals use sequential sets of tools in sophisticated ways (involving concurrent actions, holding tool and target concurrently)^22^. The flexible approach to tool related problems both in captivity as well as in the wild^13,22^ suggests that this species’ tool use behaviour is largely innovative. We define innovation here as the discovery of a new behavioural interaction with the physical environment, tapping into an existing opportunity and/or creating a new opportunity^23^.

We wanted to design an experiment that requires composite tools, like the nut cracking of Bossou's chimpanzees^4^, yet considering the bodily limitations of birds vs primates, we decided to rely on the known motor repertoire of the Goffin (such as the use of sticks or balls (e.g.^16,17,19,24^), focusing on spatial relations and not on percussive movements. Using the wording and concepts of the tooling theory, we adapted the task-related features to the organismal-structural constraints of parrots^12,25,26^. Inspired by the surroundings of the Goffin Lab (located in the Lower Austrian countryside), we designed a composite tool use task which we named the Golf Club Task.

**Figure 1:**
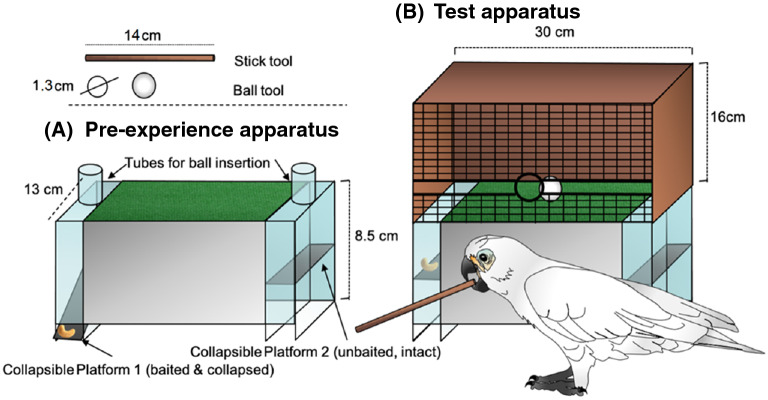
Basic apparatuses used: (**A**) Pre-experience apparatus with two insertion tubes; (**B**) Test Apparatus with frontal grid, lateral slits, and the central insertion hole.

The Golf Club Task is a puzzle box with a floor covered with carpet. The carpet is bordered by two longitudinal openings on each side, that lead to collapsible platforms, one of which is baited with a reward (Fig. 1). In order to obtain the reward, a ball has to be inserted at the front of the apparatus and thereafter pushed with a stick over the carpet into the openings containing the food. Like nut cracking, the task requires the use of two objects (in this case a ball and a stick) with different but complementary functions (second order spatial relation)^12^. Unlike in the nut cracking task, the two objects need to be moved dynamically. There must be a sequence of actions, with the ball inserted first and then the stick, and there is no constant grip on both objects, but the actor receives indirect feedback of actions on the ball (through the stick)^12^. Thus, success depends on what we propose to label an external-concurrent interaction, since the position and momentum of one object is controlled by another (pushing the ball with the stick into a specific direction). Although nut cracking requires a considerable degree of skill during aiming, the tools, the goal, and the actor, are in the same line of action, whereas in Golf Club Task the position changes dynamically during the action, and aiming drastically determines success beyond merely hitting an external object with a tool.

The organismic-structural constraints specific to avian bodies (beak-tongue-foot, rather than bimanual manipulation) are severely limiting to the study of associative tool use (see^3,27,28^, for exceptions). Thus, to this date, these types of studies are largely limited to primates^1^. As Goffin’s have already been shown to be capable of using either a ball or a stick individually to retrieve food from a puzzlebox (e.g.^19^) and of solving non-tool related multi-step sequential problems^22,29^, we predicted that individual birds would be able to find a solution to this problem, and, if they could control movement and position of the ball with the stick, they would direct it to the side with the food reward above chance expectation. The individual innovative capacities of Goffin’s^16,18,22,24,29^ makes them an ideal avian model for exploring a composite tool use task.

We additionally planned to use successful solvers as demonstrators for the remaining subjects to test whether the solution could be socially transmitted to previous non-solvers. We would find evidence of tool use emulation, as has previously been shown in non-associative tooling tasks^24^.

## Results

Out of 11 cockatoos, 3 reached the proposed criterion of 9 consecutive successful trials (2 males, 1 female), and 2 more were able to solve the Golf Club Task eventually, but without reaching criterion.

**Figure 2:**
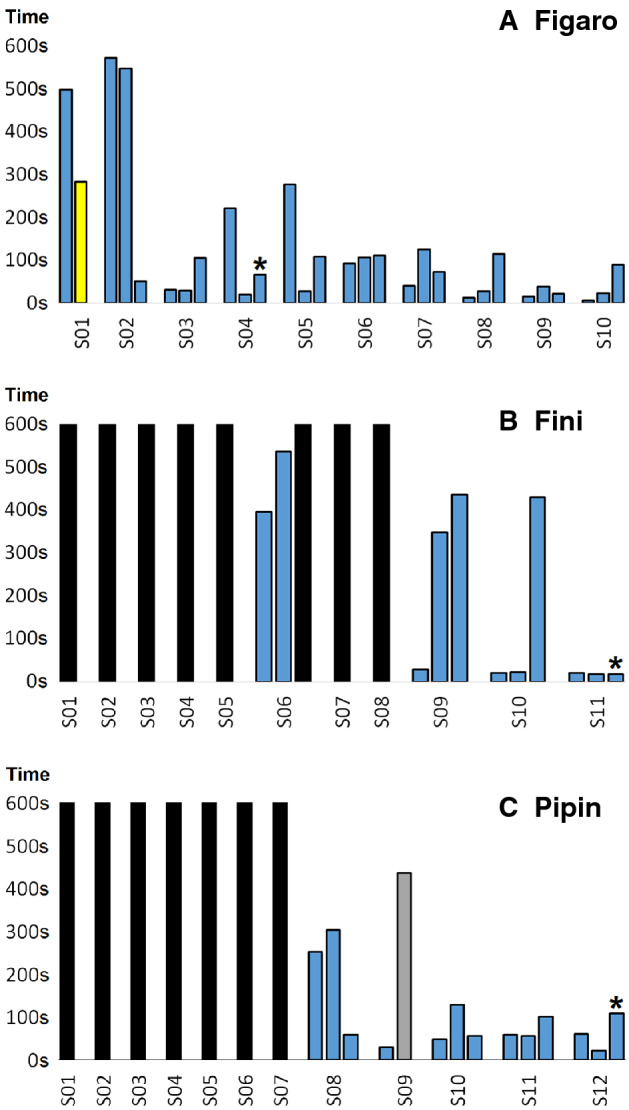
Progress until reaching criterion or completing the minimum number of sessions, for the 3 solvers: (**A**) Figaro, (**B**) Fini, and (**C**) Pipin. Colors: black (failed due to reaching the time limit), grey (failed due to inactivity), yellow (solved by cheating), and blue (solved). The asterisk marks the criterion point. Y axis, time (seconds); X axis, sessions and trials.

Among the 3 solvers, Figaro (who was also the first documented Goffin making and using tools^16^) stands out (Fig. 2A). Figaro started to solve the Golf Club Task from the first trial, and reached criterion in the fourth session (he did not reach it in the third because the second trial was cancelled as Figaro found a way to collapse the platforms by shaking the box by using the stick as a lever). After an extensive exploration of the Golf Club Task, in his first trial it took him 8:15 min to find the solution, but in the third trial of the second session he drastically reduced his success time to only 64 s. He rapidly reduced the movements necessary to solve the task, being successful on most occasions. He was able to reach a minimum time of around 5 s (e.g. 5.5 s in the first trial of the tenth session, from START to FIRST COLLAPSE). Note that the other two solvers had also a high performance history in technical problem solving task and tool use tasks^18,29^.

**Figure 3:**
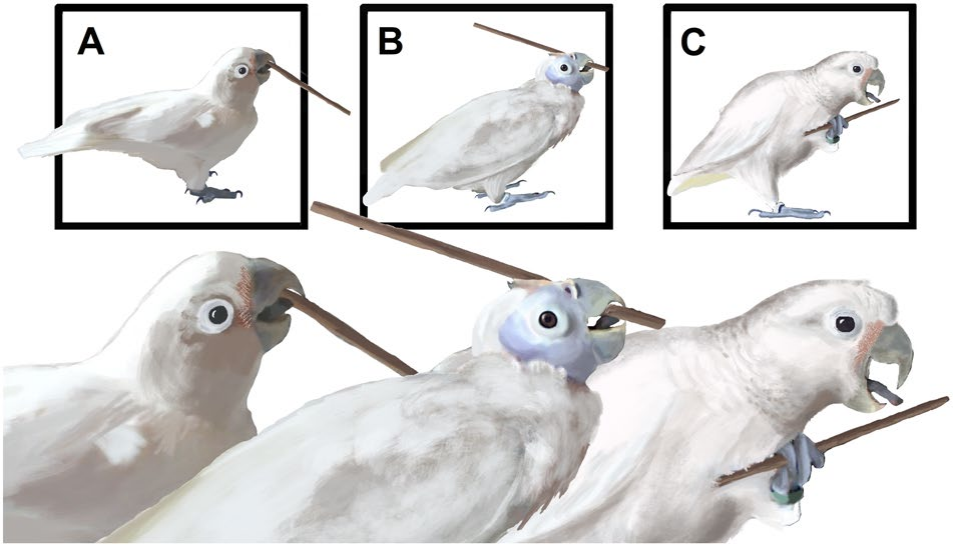
Distinctive techniques and use of the tongue during insertions for the 3 solvers: (**A**) Figaro, (**B**) Fini, and (**C**) Pipin. (**A**) Figaro used his tongue to hold the rear end of the stick towards the inner upper beak, a balance between the position of the tongue and the head allows for movements of the stick (mostly vertical); (**B**) Fini held the stick by the front end, after inserting a short portion of the stick with her head movement and used her tongue a to push the stick with repeated thrusts; (**C**) Pipin grabbed and began the insertion with a leg grip and used his tongue to guide the stick towards the goal. After completing the insertion his technique changed to a beak hold similar to (**A**).

The technique employed by the three solvers differed in three aspects (Fig. 3; Video S1): (1) the way they gripped the stick, (2) the way they inserted it, and (3) the spatial positioning (which influences where they inserted the stick and how they interacted with the ball when using it, pushing or pulling).

Despite his curved beak, Figaro griped the stick at one end, and used frontal insertions (Fig. 3A; Videos S1 and S2). This kind of grip lead to insertions that depended on a pendulum motion of his head, or even insertions during which he needed to walk forward to complete them. While inserting the stick he positioned himself close to the ball directly targeting the distal tool end at the latter, this led to the first insertion being made through the center hole of the grid.

Notably, sometimes when Figaro inserted the stick it landed on the wrong side to push the ball to the platform on which the nut was located. In those situations, Figaro lifted the stick over the ball, just enough to let it rest again on the other side of the ball, from where he could push it to send it to the correct platform. These movements required a visible effort of precision (with the beak and tongue), and of 13 instances that we observed, 10 were followed by a stroke that sent the ball to the correct platform (Video S2). This was observed also in the others solvers, but less clearly, in part due to technique differences.

The birds, particularly Figaro often repeatedly grasped and released the objects before inserting them, thereby alternating between the ball and the stick. When this happened, it was always prior to the first contact with the box (Video S1).

Pipin was the only cockatoo in the group that used his legs to grasp and insert the stick (Fig. 3). The sequence usually began with him grasping the stick with the beak (or leg in a few occasions) and thereafter switching the grip to the leg, beginning the insertion, and guiding the stick with the tongue to complete it, finishing with a grip of the stick from the end in his beak. His spatial positioning was, like Figaro's, close to the ball.

Fini, unlike the other birds, grasped the stick near the anterior end. Her grip on the stick caused body positions in which the movement of the beak seemed insufficient to complete the insertion. In those cases, she used her tongue, pushing the stick in a number of repeated thrusts to complete the action (Fig. 3B). Her spatial positioning was different from the rest of the group, usually placing herself close to the platform to be collapsed rather than in front of the ball. This favored insertions through the lateral lines of the apparatus (see Fig. 1B) and instead of pushing the ball, she thereby pulled it towards herself, ultimately using the stick in a raking rather than a poking fashion (for examples of the three different techniques used see Video S1 in the electronic supplementary material).

We recorded a few longer trial times when the birds had already achieved efficiency (Fig. 2). These extended times were sometimes caused by the birds collapsing the wrong platform, and commonly associated with problems with the structure of the test box: often the ball was left in ‘dead zones’ where it was very difficult for the cockatoos to make contact with the stick (e.g., stuck to the grid, but below the sidelines).

Nevertheless, two solvers found alternative ways to remove the ball from these dead zones. The technique used by Figaro allowed him to insert the stick with great precision. In contrast to his conspecifics, he was able to make insertions through the small mesh of the grid, below the lanes, and push the ball back into the center of the green carpet. This was immediately followed by new targeted insertions of the stick through the top lane. Fini, with a technique much less precise in her insertions, used her claws to push the ball away from the grid, and then continued with her composite tool use. She even found an alternative solution to the task by pushing the ball to the collapsible platform, using her claw (S18T2; Fig. S1).

The two cockatoos that did not reach criterion despite finding the solution at some point (Dolittle and Konrad; both males) kept on inserting the stick through the central hole of the grid without trying the lateral lanes. They solved the task only when striking the ball hard enough to get a “hole in one”. If the ball slowed down near the platform, the distance made it impossible to reach it with the club from the central hole, and required an insertion through the sidelines, which they did not attempt. Dolittle solved one trial (S06T1) and Konrad a full session (S6) with three consecutive correct trials. He also showed a similar pattern to the solvers, with a drastic drop in the time required to collapse the platform after the first trial of the first solved session (4:59 min in S09T1, to 16 s in S09T2).

Both demonstrators, Figaro and Fini, reached the aiming criterion (5 aiming errors in 20 consecutive correct trials) with only 4 aiming errors in 20 trials (totaling 25 sessions for Figaro, and 37 for Fini; which would end up being 30 and 42 after the second experiment).

Regarding the two GLMMs used to assess learning the correct tool sequence, and the ability to target using the composite tool use: the probability of collapsing the correct platform first (model 1) was not significantly affected by trial number (full-null model comparison: χ^2^ = 1.204, df = 2, P = 0.548; see the supporting information Table SI 1 for full results). Nevertheless, the probability of collapsing the correct platform first was significantly above chance (intercept: estimate ± SE = 0.716 ± 0.199, z = 3.596, P < 0.001). The probability of inserting the ball first did depend on trial number and/or its interaction with demonstrator ID (full-null model comparison: χ^2^ = 9.541, df = 2, P = 0.008). After removal of the non-significant interaction between trial number and individual (Table SI 2) we found that the probability of inserting the ball first clearly increased with trial number (Table SI 3; Fig. S3).

After having had the opportunity to observe the complete session of a solver, we noticed changes in the performance of most but not all individuals (Fig. S2). Moneypenny and Mayday (females; both Figaro observers), did not change their actions (showing disinterest in the task).

Other cockatoos showed changes in their actions, but did not show the correct sequence of movements; Heidi and Muppet (female and male; both Fini observers) damaged the box during their follow up sessions, to the point of having to stop the trials to fix it (Fig. S2). This only happened during the follow up, and not during the first experiment. It was especially evident in Heidi, whose 5 follow up sessions each had to be stopped before the time limit, and none of the previous ones were stopped (neither for inaction nor damage to the box).

Kiwi and Zozo (both males; Fini observers), also showed destructive behavior for the first time after received demonstration. Yet, more importantly they also increased the number of composite interactions between the stick and the ball substantially (Fig. S2). Kiwi showed a total of 5 composite interactions in 4 of the 5 sessions following demonstrations, having never shown any in the prior experiment. Zozo showed a total of 4 composite interactions in 3 sessions, having shown a single composite interaction in the prior experiment. After the demonstrations, Zozo collapsed the incorrect platform in the third session of the follow up, but like Kiwi, was unable to insert the stick through the lateral lines.

Dolittle’s and Konrad’s (both males; Figaro and Fini observers respectively) performance remained similar to the prior experiments (Fig. S2). They were able to solve the task during the first experiment, but were unable to do so consistently. After demonstrations they were also able to solve it (Dolittle during the first session of the follow up, and Konrad during sessions three and five), and with very short trial times (Dolittle 12.4 s in S11T1, Konrad 35.5 s in S15T1; Fig. S1), but still only when it was possible to solve through the center hole (they did not insert the stick through the lanes).

## Discussion

Our results suggests that individual Goffin’s cockatoos have the capacity to innovate the solution to a problem that requires the simultaneous use of two objects, with different but complementary functions, to achieve a single goal (composite tool^1^). 3/11 birds reached our criterion and 2 were able to solve it without reaching criterion. Those that reached criterion were able to solve the task every single time after that.

After an initial exploratory phase, the 5 successful individuals, drastically reduced their trial time, not gradually but suddenly. This suggests that the birds were able to immediately identify and memorize the necessary steps upon their initial success, a pattern that is inconsistent with typical associative learning progressions yet strongly consistent with previous Goffin problem solving performances^16–18,29^. This is especially notable if we account for the relational complexity of the task. Goffin's cockatoos possess a strong psychological drive to combine objects during play^24^ as well as during exploration and feeding, something that could eventually result in tool-use learning^13^. Perhaps the spatial relations required to solve this task are not far from those experienced during such object combinations.

The 3 cockatoos that met the criterion (Figaro and Pipin, males; Fini, female) used different techniques. Their styles differed in the way they grasped the stick, in how they inserted it, and in their spatial positioning. The grasping and insertion techniques seemed to be individually manifested upon their first tool innovation as two of the birds used similar techniques as in a previous study^24^. What might seem a trivial difference between individuals denotes the feature that makes Goffin's cockatoos an interesting model for studying innovative, non-stereotyped tooling that is likely to result from more general cognitive processing^30^. The broad spectrum of individual strategies employed by our subjects further supports the notion that the Goffin’s tooling capacities are spontaneously innovated and are based on domain general processing rather than on stereotyped, inherited behavioural routines^30–33^. This is reminiscent of the interindividual variability described in other flexible tool users such as chimpanzees, with different techniques in the use of the probe used to fish for termites in Goualougo, Republic of Congo^34^.

Although we can only speculate about it, it is likely that functional fixedness to specific tool applications (e.g.^35,36^) was the reason why two cockatoos did not reach criterion. They only inserted the stick through the central hole, not being able to innovate an insertion through the lateral lines. The efficiency and effectiveness achieved in their most advanced successful trials contrasted with the impossibility of solving the task once the ball was unreachable with the stick from the central hole. Perhaps the insertion of the stick through the lateral lines seems like a minor innovation compared to that required for solving a composite tool use task. Nevertheless, it is plausible, if we account for the amount of experience in experimental designs that include the insertion of a stick through a round central hole into an apparatus (e.g.^17,19–21^).

Figaro (probably the most assiduous tool user in our group) was able to solve the task every single time. After 3 successful but largely exploratory trials, and one in which we had to cancel the trial because he came up with an alternative solution (shaking the box by using the stick as a lever), he reduced his trial times drastically, reducing his body movements to those strictly necessary to solve the problem. His minimum trial times ranged around 5 s. In future studies we would like to compare this performance with those of young human children as they have been shown to have difficulties with certain types of tool innovations for a surprisingly long time (e.g.^37^).

The three cockatoos that reached criterion were able to adapt their movements to the different situations that the problem posed. When they did not make a ‘hole-in-one’, they followed the ball as it moved along the green, inserting the club through the lateral lines, and found different solutions to the problems intrinsic to the box design when the ball fell out of reach of the stick.

Throughout the sessions, the cockatoos demonstrated learning with respect to the order of insertions: they learned to first insert the ball and then interact with it using the stick. This first insertion of the ball into the box was sometimes preceded by a sequence of alternating grasps and releases between the ball and the stick (Video S1). This could be interpreted as a decision making process in which using the ball first should prevail over the association established in previous experiments involving the sole use of a stick (both objects have been directly associated to food in previous experiments, e.g.^19,21^, and more unpublished studies), as well as the success using it during previous trials. This cyclical movement, picking up and dropping objects, when a decision has to be made, is something that has been observed in previous experiments with Goffin's cockatoos^20,38^, and also in kea (*Nestor notabilis*; Megan Lambert personal communication). Within the course of several sessions the insertion of the ball was established as the default first action (Table SI 3; Fig. 1).

Perhaps one of the most relevant results is that the cockatoos were not only able to innovate the sequential and simultaneous use of two objects with different but complementary functions, but that they are able to aim by composite tool use. Although we did not observe an improvement in their targeting ability throughout the experiment (Table SI 1), they did aim at the correct platform above chance.

Being able to aim during the use of a tool set can be very important, even in actions with arguably simpler spatial relationships, as for example in primate nut-cracking^12,39–41^. It is relatively common to find capuchin monkeys with amputations as a result of errors during nut-cracking^11^). Aiming is also essential in the more complex forms of composite tools used by humans, and in the sport that inspired our experimental design^7^. Golf, like other sporting activities in which composite interaction between objects is employed, probably owes its playful activity to the difficulty involved in aiming behaviour.

The spatial relations required to solve this task by aiming, as the cockatoos have shown, involve complex relations between the two objects and the actor. The specific spatial orientation of the sequential object insertions was followed by an alignment of the stick, with the ball towards the baited collapsible platform in a dynamic directed and allocentric spatial relation. The aiming success illustrates the control of the cockatoos over this action, and can further be highlighted by corrective actions performed in certain occasions to adjust the position of the stick (Video S2). We describe the control of the ball position through the stick as an external-concurrent interaction. To our knowledge the only case of nonhuman animals managing concurrent relations while holding two tools at the same time is the chimpanzee’s nut cracking while holding the anvil and the hammer (as occurs on the rare occasions when it is performed on a tree branch; Boesch and Boesch, 1993, and 2017). Birds have obvious physical limitations in grasping and using two tools at the same time, and this external-concurrent interaction may be as close as they can come to achieving such an action. Their limitations seem to be related to their body plan, not dexterity, as shown by the recent discovery of wild Goffin’s handling a single tool (stick) and the target (fruit) in a concurrent manner^22^.

In order to explore the ability to socially transmit composite tool use, we conducted a second experiment in which the cockatoos were divided into observers (those that had not reached a criterion during the first experiment) and demonstrators (two solvers chosen for this task). The results of this follow-up should be taken with caution, because being an extension of the previous experiment, we cannot be fully sure that the changes reflected in the observers were a product of having received a demonstration, or simply delayed learning due to the accumulated number of sessions. In any case, the changes are interesting and, in some cases, drastic enough to suggest that the demonstrations may have affected their performance. In fact, the results suggest abrupt improvement in the performance of some males, but not in females. The sex of demonstrator could affect the attention of the observers, as has been shown in other species^42,43^, but due to our small sample this has to remain a target for future studies. The result of this experiment is coherent with our current understanding of their emulation capabilities, and lack of imitation regarding tool use^24^.

Composite interactions and destructive behavior towards the box increased in observers, in some cases drastically after the first demonstration (Fig. S2). This could be explained by the frustration caused by viewing a task being solved by another individual, but we also cannot rule out the similarly frustrating effect of accumulating unsuccessful sessions. Interestingly, the techniques used by the demonstrator did not match the observers. For example, Kiwi and Zozo, drastically increased their composite interactions, but as mentioned before, only inserted the stick through the central hole. Nevertheless, Fini (their demonstrator) used a very different technique, based on a spatial positioning close to the platform to be collapsed and then inserting the stick along the sidelines. This parallels previous findings suggesting that Goffin’s learned by emulation, not imitation^24^.

In conclusion, the first Golf Club Task experiment shows that some individual Goffin’s are able to innovate the solution to a task that requires the use of two objects with complementary functions and complex spatial relationships, they learned the sequence to use a set of tools, and they were able to aim using composite tool use^1^. The second experiment, although very limited, provided tentative support of social learning by emulation.

To our knowledge, this is the first example of the combination of these abilities outside primates and indicates that the cognitive preconditions for composite tool use have also evolved outside the primate lineage.

## Materials and methods

### Subjects

Eleven adult cockatoos (7 males, 4 Females; all adults, hatched between 2007 and 2011) took part in this experiment. All the Goffin Lab’s birds are captive born and purchased from accredited European breeders, have full CITES certificates and are officially registered (following the Austrian Animal Protection Act § 25—TschG. BGBl. 118) at the district’s administrative animal welfare bureau (Bezirkshauptmannschaft St. Pölten Schmiedgasse 4–6, A-3100; St. Pölten, Austria). They are permanently housed under environmentally and socially enriched conditions at the Goffin Lab in Goldegg (Lower Austria), with an indoor (45 m^2^; 3–6 m high) and outdoor (ca. 200 m^2^; 3–4.5 m high) aviary. The birds are provided with a varied and mostly fresh diet, and a minimum temperature of 17ºC during the winter, with 12:12 light and dark cycles. All birds were adapted to a daily experimental routine and observation, and the 11 birds used in this study have tooling experience from previous studies, including several stick and ball tools^16–19,19,24^, but none in which both objects had to be combined simultaneously to achieve the same goal.

### Ethics

Since the presented experiments are purely appetitive and strictly non-invasive they do not qualify as ‘Animal Experiments’ according to Austrian Law, nevertheless, this study was approved by our institutions ethics and animal welfare committee (University of Veterinary Medicine, Vienna) in accordance with good scientific practice guidelines and national legislation within WWTF project number CS18-023. All animals were purchased from European breeders in accordance with CITES regulations, and are registered at the local Animal Welfare Bureau (Bezirkshauptmanschaft St Pölten, NÖ). None of the birds are clipped, and all participated in our experiments on a voluntary basis (when a bird is required for testing it is called by name to come to the experimenter’s hand).

### Apparatuses

Our Golf Club Task apparatus is a largely transparent box composed of two parts: a Plexiglas base with a rough (carpet-like) central "green" (see Fig. 1B). The green is located between two collapsible platforms, one on each side. The platforms are held in place with magnets. The top part is a wooden structure (which is firmly fixed onto the base). It has a frontal grid with a round central hole and two open lines on both sides of it (narrower than the diameter of the central hole). In each trial, 1/8 of a cashew nut is placed on one of the two collapsible platforms. Alongside the apparatus we provided subjects with a set of two tools to solve the problem: a white ball (big enough to fit through the hole but not through the lanes or the grid; 10 g and 1.3 cm thick) and a wooden stick (14 cm long and 0.6 cm wide axis; 3 g).

Prior to testing, the cockatoos received information on the collapsibility of the two platforms by receiving pre-experience during which they were able to drop a heavy object (with a black, irregular, and plastic surface; 5 g and 1.8/1 cm as maximum/minimum lengths) on one of them. The basic apparatus was the same as used for testing but the upper structure of the box was removed and an extra Plexiglas surface was added over the platforms limiting the possibility of collapsing the platforms to only two transparent tubes (Fig. 1A). During this pre-experience phase subjects could retrieve the food by inserting the small ball into one of the tubes.

### Procedure

#### Pre-experience

As mentioned before, the birds had experimental experience with ball and stick type tools in several different contexts (e.g.^19,21^). Thus, they received a single session with 5 consecutive trials of 5 min. maximum. The position of the nut on one or another platform varied randomly between trials.

#### Testing

Subjects were tested (on the complete Golf Club box) over a minimum of 10 sessions for a maximum of 3 trials each. If an individual reached the time limit (10 min), the trial was scored as failed, the session was terminated and the cockatoo tested again on another day. If the cockatoo collapsed the wrong platform, the ball could be retrieved through the lower opening and reused within the available time limit. We used a criterion of 3 consecutive correct sessions to consider a cockatoo a consistent solver (as each session is composed of 3 different trials, to reach criterion they need to solve 9 consecutive trials). If a cockatoo had started solving in the ninth or tenth session, it was allowed up to 2 more sessions to reach criterion. The position of the stick and ball was randomized in each trial (left or right), as was the position of the nut, which could be found on either platform (this was done using random.org).

After the first experiment non-solvers received demonstrations from solvers. We extended the number of sessions for two of the solvers (one male and one female that were socially compatible with the observers) to a new criterion in order to achieve a level of accuracy for the demonstrations: collapsing the correct platform first with only 5 errors in 20 trials solved consecutively (note that here "error" means having collapsed the wrong platform first, not having failed the trial). After reaching the criterion, these two birds became demonstrators. The observers were divided into two groups, with each individual assigned to one of the two demonstrators. Due to social incompatibilities between some cockatoos, the observers had to be unevenly distributed, leaving 3 observers for Figaro (1 male, Dolittle; 2 females, Mayday and Moneypenny) and 5 for Fini (4 males, Zozo, Kiwi, Konrad, Muppet; 1 female, Heidi). The observers had the opportunity to witness a demonstrator solve the task for a full session (3 Trials) before they were confronted with the apparatus themselves. For each observer, we conducted 5 sessions with a maximum of 3 trials (10 min). The set up was the same, but with an observer located inside a cage at 1.5 m of distance and elevated enough (on a perch) to have full view of the demonstrator’s actions.

If a bird did not interact with the box (direct contact or using the objects) for 2 min, the trial was ended (the minimum time per trial if birds did not interact was 3:30 min). Similarly, if the box was damaged in a way that required immediate repair, the trial was ended for the day.

### Analysis

The pre-experiment was videotaped and coded in situ, as well as from the videos. Test trials were recorded using two cameras (JVC GZ-HM30 and Sony NEX-5), one on either side, to get a good perspective irrespective of where the cockatoo was located. Like the pre-experiment, the test was coded in situ as well as from the videos using a video coding software (BORIS 7.4.3). We recorded the time taken per trial, the collapsed platforms (baited or unbaited platform), the number of composite interactions between both objects, and the cockatoos’ interactions with the ball and the stick (frequency of contacts and insertions), as well as with the box (interaction using the tongue or fingers with the ball through the grid, and destructive behavior).

To test whether the subjects who solved the task learned to use the tool set in the correct sequence, and whether they were actually able to target the baited platform with the stick-plus-ball system above chance, we conducted two Generalized Mixed Models (GLMM^44^). We analyzed the performance of the three solvers during the first experiment, and included in the analysis the extended experience of the second experiment (while acting as demonstrators) for two of them.

With the first model (model 1) we estimated the extent to which the probability of collapsing the correct platform first was influenced by the identity of the successful bird, the trial number, and which platform was the correct one (left or right). Since we reasoned that a possible increase of this probability could depend on the successful bird, we also included the interaction between trial number and individual. We fitted a Generalized Linear Mixed Model (GLMM^44^) with binomial error structure and logit link function^45^ with all the above mentioned terms included as fixed effects. To avoid pseudo-replication due to having repeated observations of the same individuals, we included individual as a random intercepts effect, and to avoid an overconfident model and keep type I error rate at the nominal level of 0.05 we included random slopes^46,47^ of trial number, the individual as well as correct box (the latter two manually dummy coded and then centered) into the model. Originally, we included also parameters for the correlations among the random intercepts and slopes, but since all absolute correlation parameters were estimated to be essentially 1 which is indicative of them not being identifiable^48^, we excluded them from the model. As an overall test of effect of trial number and its interaction with individuals and to avoid 'cryptic multiple testing'^49^ we compared this full model with a null model lacking trial number and its interaction with individual in the fixed effects part but being otherwise identical. With essentially the same model we also tested whether the probability to collapse the correct platform first was on average above chance. To this end, we manually dummy coded and then centered individual and correct box to a mean of zero and then fitted the same model as described above. In this model, the intercept tests the hypothesis that the average proportion of trials in which individuals collapsed the correct platform first is at chance level.

With the second model (model 2) we estimated what influenced the probability to insert the ball first. This model was identical to model 1 with the exception that this time we could also include a random slope of interaction between trial number and its interaction with individual. Again, we excluded the parameters for the correlations among the random intercept and slopes as these were all estimated to be essentially 1 or − 1.

We fitted both models in R (version 4.0.3; R Core Team. 2020^50^) using the function glmer of the package lme4 (version 1.1-25^51^) and using the optimizer bobyqa. We determined model stability by dropping the individuals from the data, one at a time, fitting the full model to each of the subsets and comparing the range of model estimates obtained with those obtained for the full data set. This revealed the models to be in part very unstable, likely due to complete separation^52^ (see the supporting information). We estimated confidence intervals of model coefficients by means of a parametric bootstrap (N = 1000 bootstraps; function bootMer of the package lme4). We tested the significance of individual effects by dropping them from a model, one at a time, and comparing the respective reduced models with the full model. All model comparisons were based on likelihood ratio tests^53^. The samples analysed for the two models comprised a total of 216 trials (in 142 of which the correct platform was collapsed first) conducted with 5 individuals (model 1) and 243 trials (in 222 of which the ball was inserted first) conducted with 5 individuals. Due to rarity of insertions of the stick first model 2 likely has low power.

## Supplementary Information

Supplementary Information.

Supplementary Video 1.

Supplementary Video 2.
