# Supplementary material for: Innovative composite tool use by Goffin’s cockatoos (Cacatua goffiniana)
Source: Sci Rep. 2022 Jan 27;12:1510. doi: 10.1038/s41598-022-05529-9 (PMC8795444; doi:10.1038/s41598-022-05529-9)
Supplement: Supplementary file 1 — Supplementary Information. [file 41598_2022_5529_MOESM1_ESM.pdf]

Innovative composite tool use by Goffin's cockatoos (*Cacatua goffiniana*).

Antonio J. Osuna-Mascaró[1], Roger Mundry[1,2], Sabine Tebbich[3], Sarah R. Beck[4], Alice M.I. Auersperg[1]

1 Messerli Research Institute, Veterinärplatz 1, 1210, Vienna., University of Veterinary Medicine, Medical University of Vienna & University of Vienna

2 Platform Bioinformatics and Biostatistics, VetMedUni, Vienna; currently at Cognitive Ethology Laboratory, German Primate Center, Leibniz Institute for Primate Research, Kellnerweg 4, 37077 Göttingen; Department for Primate Cognition, Georg-August-University Göttingen, Germany; and Leibniz ScienceCampus Primate Cognition, Göttingen, Germany

3 Department of Behavioural Biology, University of Vienna, Althanstraße 14,1090, Vienna, Austria

4 School of Psychology, University of Birmingham, Edgbaston, Birmingham, B15 2TT, UK.

Author for correspondence:

Antonio J. Osuna-Mascaró

e-mail: Antonio.OsunaMascaro@vetmeduni.ac.at

Supporting Information

Video Legends

Video S1. The technique employed by the three solvers, Figaro, Fini and Pipin, differing in: the way of gripping the stick, insertion method, spatial positioning, and pushing or pulling actions. Full speed and slow motion.

Video S2. A precision strike by Figaro, and the subsequent collapse of the baited platform. Full speed and slow motion, to appreciate the subtle movements (of beak, tongue, and head) required to complete the action.

Figures

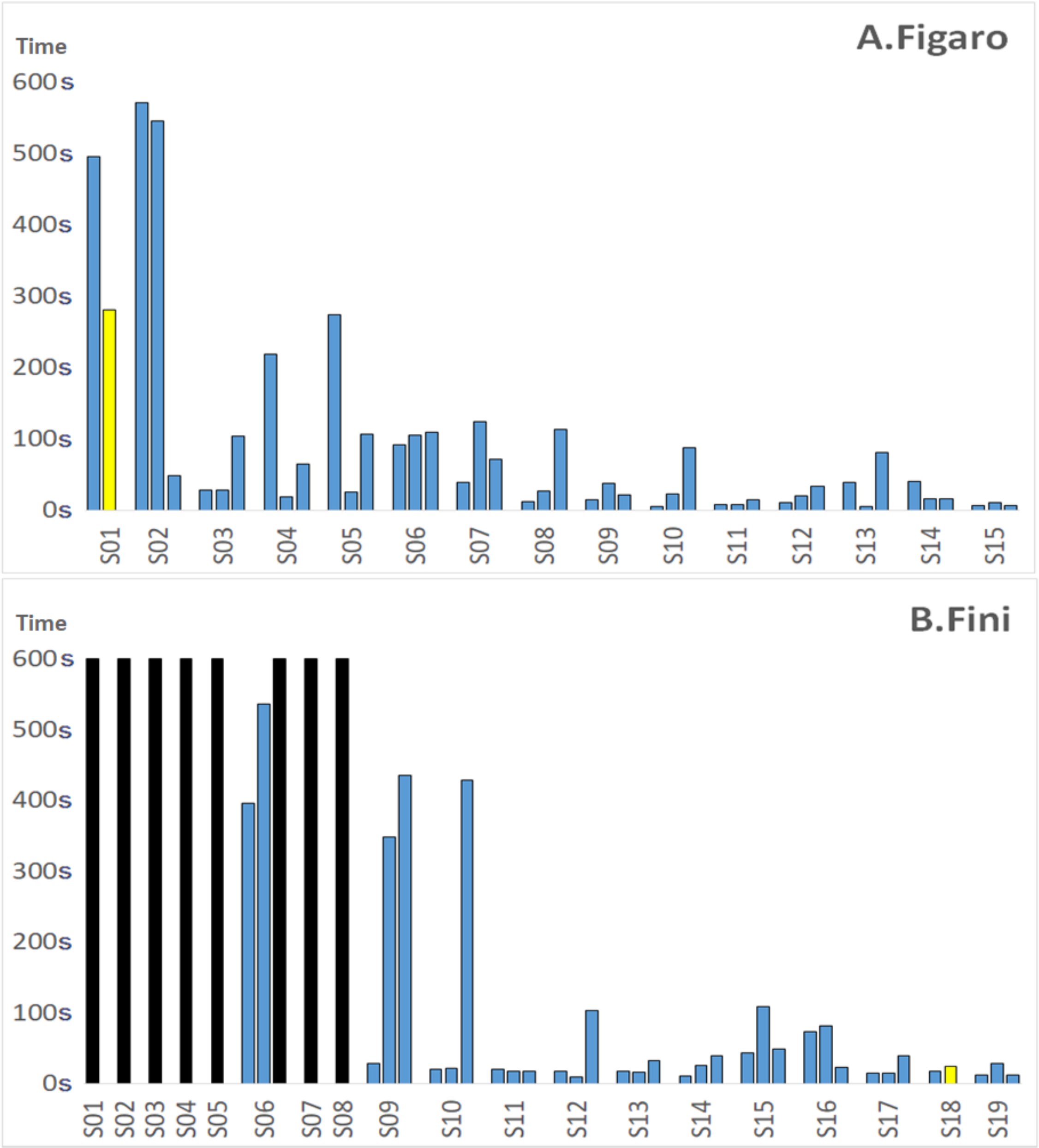

Figure S1. Performance for Figaro (A) and Fini (B). Colors: Colors: black (failed by time limit), yellow (solved by cheating), and blue (solved). Y axis, time (seconds); X axis, sessions and trials.

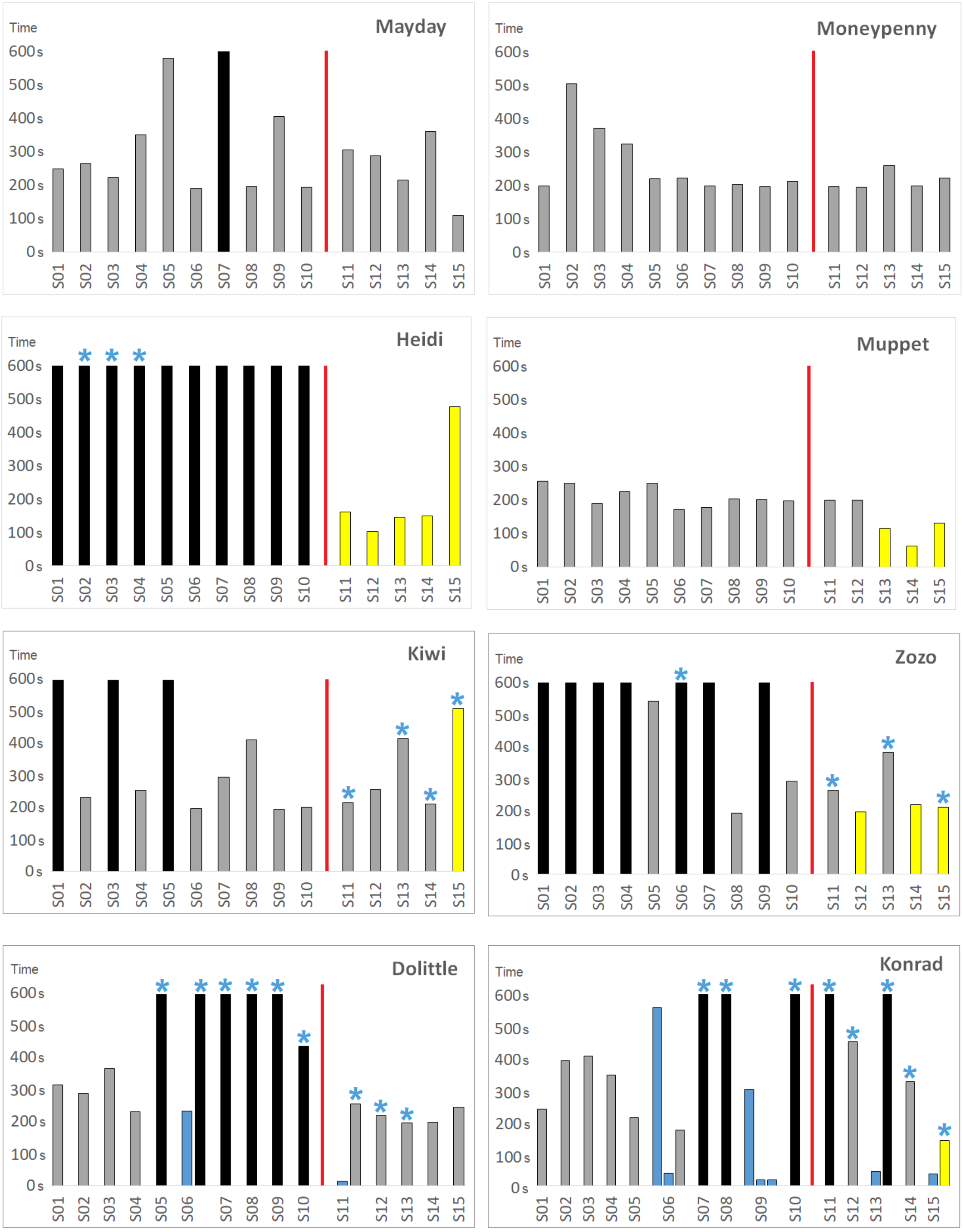

Figure S2. Performance of non-solvers during both experiments. Colors: black (failed due to time limit reached), grey (failed due to inactivity), yellow (solved by cheating), and blue (solved). The blue asterisk marks unsuccessful trials with composite interactions. A red vertical line separates session 10 from 11,sessions 1-10 belong to the innovation test, 11-15 to the social learning test. Y axis, time (seconds); X axis, sessions and trials.

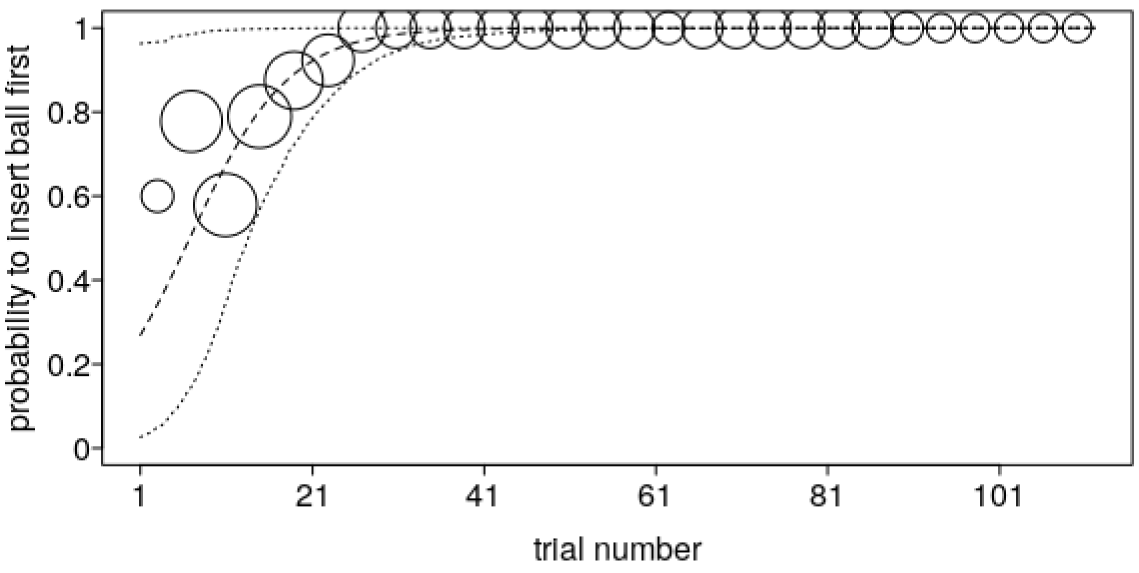

Figure S3. Probability to insert the correct tool first as a function of trial number. The dots show the probability per bin of trial number, whereby their area is proportionate to the number trials per bin (range: 4 to 19). The dashed and dotted lines depict the fitted model and its confidence limits with individual and side of the box manually dummy coded and then centered.

Tables

Table SI 1. Results of the fixed effects part of the model with collapsing the correct platform first as the response (model 1; estimates, together with standard errors, confidence limits, significance tests, and range of model estimates obtained when dropping individuals from the data, one at a time).

term	Estimate	SE	lower Cl	upper Cl	χ^2	df	P	min	max
intercept	2.011	0.879	0.269	4.137			(1)	0.701	2.261
individual(2)	-0.844	0.744	-2.602	0.558			(1)	-1.127	0.939
trial nr.(3)	0.571	0.682	-0.754	2.092			(1)	-1.518	0.846
box(4)	-0.978	0.962	-2.910	0.880	1.107	1	0.293	-1.584	-0.014
individual:trial nr.	-0.779	0.744	-2.473	0.669	1.107	1	0.293	-1.908	1.393

(1) not indicated because of being of very limited interpretability

(2) dummy coded with 'no' being the reference category

(3) z-transformed to a mean of zero and a standard deviation of one; mean and standard deviation of the original variable were 48.778 and 30.149, respectively

[4] dummy coded with 'left' being the reference category

Table SI 2. Results of the fixed effects part of the full model with inserting the ball first as the response (model 2; estimates, together with standard errors, confidence limits, significance tests, and range of model estimates obtained when dropping individuals from the data, one at a time).

term	Estimate	SE	lower Cl	upper Cl	χ^2	df	P	min	max
intercept	7.656	1.845	4.790	15.650			[1]	6.265	9.176
individual[2]	-0.378	3.814	-6.690	152.146			[1]	-1.010	15.777
trial nr.[3]	5.144	1.479	2.688	10.991			[1]	4.107	6.228
box[4]	-0.866	0.574	-2.807	0.410	2.335	1	0.127	-1.102	-0.591
individual:trial nr.	1.470	4.240	-4.143	163.345	0.154	1	0.695	-4.848	12.288

[1] not indicated because of being of very limited interpretability

[2] dummy coded with 'no' being the reference category

[3] z-transformed to a mean of zero and a standard deviation of one; mean and standard deviation of the original variable were 44.490 and 30.950, respectively

[4] dummy coded with 'left' being the reference category

Table SI 3. Results of the fixed effects part of the reduced model (lacking the interaction between individual and trial number) with inserting the ball first as the response (model 2; estimates, together with standard errors, confidence limits, significance tests).

term	Estimate	SE	lower CI	upper CI	χ^2	df	P
intercept	7.872	1.764	5.315	16.468			(1)
individual(2)	-1.645	0.957	-4.642	4.687	2.607	1	0.106
trial nr.(3)	5.368	1.375	3.124	11.618	9.387	1	0.002
box(4)	-0.868	0.574	-2.579	0.383	2.351	1	0.125

(1) not indicated because of being of very limited interpretability

(2) dummy coded with 'no' being the reference category

(3) z-transformed to a mean of zero and a standard deviation of one; mean and standard deviation of the original variable were 44.490 and 30.950, respectively

(4) dummy coded with 'left' being the reference category

Table SI 4. Results of the random effects part of the model with collapsing the correct platform first as the response (model 1; estimated standard deviations, together with their range obtained when dropping individuals from the data, one at a time).

effect(1)	sd	min	max
intercept	0.223	<0.001	0.249
individual(2)	0.000	<0.001	<0.001
trial nr.(2)	0.000	<0.001	<0.001
box(3)	1.615	0.723	2.307

(1) 'intercept' denotes a random intercepts effect, all others a random slope

(2) dummy coded and then centered

(3) z-transformed to a mean of zero and a standard deviation of one; mean and standard deviation of the original variable were 48.778 and 30.149, respectively

Table SI 5. Results of the random effects part of the model with inserting the ball first as the response (model 2; estimated standard deviations, together with their range obtained when dropping individuals from the data, one at a time).

effect[1]	sd	min	max
intercept	1.081	<0.001	1.566
individual[2]	0.000	<0.001	<0.001
trial nr.[3]	0.000	<0.001	1.143
box[2]	0.000	<0.001	<0.001
individual:trial nr.	0.000	<0.001	0.921

(1) 'intercept' denotes a random intercepts effect, all others a random slope

(2) dummy coded and then centered

(3) z-transformed to a mean of zero and a standard deviation of one; mean and standard deviation of the original variable were 44.490 and 30.950, respectively
